# Supplementary material for: Record linkage studies of drug-related deaths among former adult prisoners who have been released to the community: a scoping review protocol
Source: BMJ Open. 2022 Mar 29;12(3):e056598. doi: 10.1136/bmjopen-2021-056598 (PMC8966574; doi:10.1136/bmjopen-2021-056598)
Supplement: Supplementary data [file bmjopen-2021-056598supp001.pdf]

Record linkage studies of drug-related deaths among former adult prisoners who have been released to the community: a scoping review protocol

Cooper JA [1,2]*, Onyeka IN [1,2], O'Reilly D [1,2], Kirk R [3], Donnelly M [1,2]

[1] Centre for Public Health, Queen's University Belfast, Royal Hospitals Site, Grosvenor Road, Belfast, UK

[2] Administrative Data Research Centre Northern Ireland (ADRC NI), Centre for Public Health, Queen's University Belfast, Royal Hospitals Site, Grosvenor Road, Belfast, UK

[3] South Eastern Health and Social Care Trust, Ulster Hospital, Dundonald, UK

*corresponding author

Supplementary material

Appendix 1

Ovid MEDLINE(R) ALL <1946 to August 17, 2021>

1 Prisoners/ 17290

2 ex-prisoner*.mp. 201

3 former prisoner*.mp. 262

4 inmate*.mp. 5441

5 ex-inmate*.mp. 17

6 former inmate*.mp. 102

7 Criminals/ 5440

8 ex-criminal*.mp. [mp=title, abstract, original title, name of substance word, subject heading word, floating sub-heading word, keyword heading word, organism supplementary concept word, protocol supplementary concept word, rare disease supplementary concept word, unique identifier, synonyms] 0

9 former criminal*.mp. 0

10 convict*.mp. 7081

11 ex-convict*.mp. 12

12 former convict*.mp. 3

13 detainee*.mp. 989

14 ex-detainee*.mp. 8

15 former detainee*.mp. 8

16 Prisons/ 10227

17 Jails/ 66

18 gaol*.mp. 150

19 imprison*.mp. 2645

20 incarcerat*.mp. [mp=title, abstract, original title, name of substance word, subject heading word, floating sub-heading word, keyword heading word, organism supplementary concept word, protocol supplementary concept word, rare disease supplementary concept word, unique identifier, synonyms] 12802

21 detention*.mp. 3423

22 correction*.mp.235912

23 confinement.mp. 22002

24 Correctional Facilities/ 69

25 penal institution*.mp. 133

26 penitentiar*.mp. [mp=title, abstract, original title, name of substance word, subject heading word, floating sub-heading word, keyword heading word, organism supplementary concept word, protocol supplementary concept word, rare disease supplementary concept word, unique identifier, synonyms] 693

27 remand.mp. 295

28 offender*.mp. 12056

29 ex-offender*.mp. 102

30 former offender*.mp. 4

31 after release.mp. 4226

32 prison release.mp. 136

33 released prisoner*.mp. 134

34 following release.mp. 1117

35 recently released.mp. 1911

36 newly released.mp. 786

37 postrelease.mp. 341

38 post-release.mp. 787

39 liberat*.mp. 38930

40 "Cause of Death"/ or Death/ 69103

41 Mortality/ 47259

42 Fatal Outcome/ 65463

43 fatal*.mp. 214521

44 lethal*.mp. 163790

45 Substance-Related Disorders/ 99086

46 Opioid-Related Disorders/ 17378

47 drug abuse*.mp. 21345

48 drug dependen*.mp. 6424

49 Drug Misuse/ or Prescription Drug Misuse/ or Substance Abuse, Intravenous/ 18137

50 Drug Overdose/ 12514

51 drug poisoning.mp. 705

52 Drug Users/ or Injections, Intravenous/ 85588

53 substance abuse*.mp. 55910

54 substance dependen*.mp. 3072

55 substance misuse*.mp. 3022

56 substance use*.mp. 42442

57 Illicit Drugs/ 11763

58 substance poisoning.mp. 17

59 street drug*.mp. 735

60 toxicity.mp. 750642

61 Opiate Overdose/ or Analgesics, Opioid/ 49884

62 Heroin Dependence/ or Heroin/ 13437

63 drug withdrawal.mp. 3932

64 accidental poisoning.mp. 912

65 Benzodiazepines/ 22377

66 Cocaine-Related Disorders/ or Cocaine/ or Crack Cocaine/ or Cocaine Smoking/ 30584

67 Narcotics/ 16548

68 1 or 2 or 3 or 4 or 5 or 6 or 7 or 8 or 9 or 10 or 11 or 12 or 13 or 14 or 15 or 16 or 17 or 18 or 19 or 20 or 21 or 22 or 23 or 24 or 25 or 26 or 27 or 28 or 29 or 30 or 31 or 32 or 33 or 34 or 35 or 36 or 37 or 38 or 39 353126

69 40 or 41 or 42 or 43 or 44 475646

70 45 or 46 or 47 or 48 or 49 or 50 or 51 or 52 or 53 or 54 or 55 or 56 or 57 or 58 or 59 or 60 or 61 or 62 or 63 or 64 or 65 or 66 or 67 1110035

71 68 and 69 and 70 530

72 limit 71 to english language 473
