# Supplementary material for: Record linkage studies of drug-related deaths among former adult prisoners who have been released to the community: a scoping review protocol
Source: BMJ Open. 2022 Mar 29;12(3):e056598. doi: 10.1136/bmjopen-2021-056598 (PMC8966574; doi:10.1136/bmjopen-2021-056598)
Supplement: Supplementary data [file bmjopen-2021-056598supp002.pdf]

Record linkage studies of drug-related deaths among former adult prisoners who have been released to the community: a scoping review protocol

Cooper JA [1,2]*, Onyeka IN [1,2], O'Reilly D [1,2], Kirk R [3], Donnelly M [1,2]

[1] Centre for Public Health, Queen's University Belfast, Royal Hospitals Site, Grosvenor Road, Belfast, UK

[2] Administrative Data Research Centre Northern Ireland (ADRC NI), Centre for Public Health, Queen's University Belfast, Royal Hospitals Site, Grosvenor Road, Belfast, UK

[3] South Eastern Health and Social Care Trust, Ulster Hospital, Dundonald, UK

*corresponding author

DATA CHARTING FORM

**Study assessor initials**		
**Date of completion of data charting form**		
**Item**	***Notes on item***	**Data extracted**
**Study reference number**		
**Study assessment number**		
**Lead author**		

**Title**		
**Year of publication**		
**Origin, country of origin**		
**Journal name**		
**Study design**		
**Citation**		
**Setting**	*Stated setting*	
	Stated locations	
	Stated relevant dates	
**Participants**	*Stated study population*	
	Stated type of prison	
	Stated age inclusions (or any stated age exclusions)	
	Stated gender inclusions	
	Stated race/ethnicity inclusions	
	Stated methods of study population selection (type of prisoner data records)	
	Stated methods of follow-up (for example, record linkage or interview)	

	Stated rationale for the choice of cases and controls (case-control study only)	
	Stated matching criteria (case-control study only)	
	Stated number of controls per case (case-control study only)	
**Data sources**	*Stated sources of data*	
	Stated sources of comparator data	
**Bias**	*Stated bias*	
	Stated efforts to address potential sources of bias	
	Stated methods for dealing with repeated incarcerations?	
**Study size**	*Stated study size*	
**Statistical methods**	*Stated statistical methods*	
	Stated methods controlling for confounders and methods for examining subgroups and interactions	
	Stated sensitivity analyses	
	Stated time period examined after prison release	
	Stated how missing data was addressed	

	Stated how loss to follow-up was addressed	
	Stated how matching of case and controls was addressed (case-control study only)	
**Linkage**	*Stated linkage i.e. if person-level, institution-level or other data linkage across two or more databases*	
	Stated types of linked databases	
	Stated methods of linkage	
**Main outcomes**	*Stated outcome events or summary measures*<br>*(include information on specific drugs)*	
**Main results**	*Stated unadjusted estimate and, if applicable, confounder adjusted estimates, their precision, which confounders were adjusted for.*<br><br>*Reported for all cause and drug-related deaths*<br><br>*For example, include crude mortality rates, standardised mortality ratios etc.*<br><br>*Include reported for age, gender, race/ethnicity etc*	

	Stated age at release (mean/median/SD/range)	
	Stated length of incarceration (mean/median/SD/range)	
**Limitations**	*Stated limitations of study including sources of potential bias or imprecision (are the results generalisable)*	
**Quality assessment checklist or technique**	*Stated if quality assessment checklist or technique was used*	
