# Supplementary material for: Record linkage studies of drug-related deaths among former adult prisoners who have been released to the community: a scoping review protocol
Source: BMJ Open. 2022 Mar 29;12(3):e056598. doi: 10.1136/bmjopen-2021-056598 (PMC8966574; doi:10.1136/bmjopen-2021-056598)
Supplement: Author's manuscript [file bmjopen-2021-056598.draft_revisions.pdf]

BMJ Open

BMJ Open is committed to open peer review. As part of this commitment we make the peer review history of every article we publish publicly available.

When an article is published we post the peer reviewers' comments and the authors' responses online. We also post the versions of the paper that were used during peer review. These are the versions that the peer review comments apply to.

The versions of the paper that follow are the versions that were submitted during the peer review process. They are not the versions of record or the final published versions. They should not be cited or distributed as the published version of this manuscript.

BMJ Open is an open access journal and the full, final, typeset and author-corrected version of record of the manuscript is available on our site with no access controls, subscription charges or pay-per-view fees (http://bmjopen.bmj.com).

If you have any questions on BMJ Open's open peer review process please email info.bmjopen@bmj.com

BMJ Open

Record linkage studies of drug-related deaths among former adult prisoners who have been released to the community: a scoping review protocol

Journal:	*BMJ Open*
Manuscript ID	bmjopen-2021-056598
Article Type:	Protocol
Date Submitted by the Author:	23-Aug-2021
Complete List of Authors:	Cooper, Janine; Queen's University Belfast, Centre for Public Health, Queen's University Belfast, Royal Hospitals Site, Grosvenor Road, Belfast, UK; Queen's University Belfast, Administrative Data Research Centre Northern Ireland (ADRC NI), Centre for Public Health, Queen's University Belfast, Royal Hospitals Site, Grosvenor Road, Belfast, UK<br>Onyeka, Ifeoma ; Queen's University Belfast, Centre for Public Health, Queen's University Belfast, Royal Hospitals Site, Grosvenor Road, Belfast, UK; Queen's University Belfast, Administrative Data Research Centre Northern Ireland (ADRC NI), Centre for Public Health, Queen's University Belfast, Royal Hospitals Site, Grosvenor Road, Belfast, UK<br>OReilly, Dermot; Queen's University Belfast, Centre for Public Health, Queen's University Belfast, Royal Hospitals Site, Grosvenor Road, Belfast, UK; Queen's University Belfast, Administrative Data Research Centre Northern Ireland (ADRC NI), Centre for Public Health, Queen's University Belfast, Royal Hospitals Site, Grosvenor Road, Belfast, UK<br>Kirk, Richard; South Eastern Health and Social Care Trust, Ulster Hospital, Dundonald, UK<br>Donnelly, Michael; Queen's University Belfast, Centre for Public Health, Queen's University Belfast, Royal Hospitals Site, Grosvenor Road, Belfast, UK; Queen's University Belfast, Administrative Data Research Centre Northern Ireland (ADRC NI), Centre for Public Health, Queen's University Belfast, Royal Hospitals Site, Grosvenor Road, Belfast, UK
Keywords:	EPIDEMIOLOGY, MENTAL HEALTH, PUBLIC HEALTH

SCHOLARONE™
Manuscripts

Record linkage studies of drug-related deaths among former adult prisoners who have been released to the community: a scoping review protocol

Cooper JA [1,2]*, Onyeka IN [1,2], O'Reilly D [1,2], Kirk R [3], Donnelly M [1,2]

[1] Centre for Public Health, Queen's University Belfast, Royal Hospitals Site, Grosvenor Road, Belfast, UK

[2] Administrative Data Research Centre Northern Ireland (ADRC NI), Centre for Public Health, Queen's University Belfast, Royal Hospitals Site, Grosvenor Road, Belfast, UK

[3] South Eastern Health and Social Care Trust, Ulster Hospital, Dundonald, UK

*corresponding author

Corresponding author contact: Cooper JA, Centre for Public Health, Queen's University Belfast, Royal Hospitals Site, Grosvenor Road, Belfast, UK. Administrative Data Research Centre Northern Ireland (ADRC NI), Centre for Public Health, Queen's University Belfast, Royal Hospitals Site, Grosvenor Road, Belfast, UK. Email: j.cooper@qub.ac.uk

Abstract

Introduction

Prisoners often have a complex mix of health and care needs including needs relating to mental and physical ill-health conditions. In addition, substance use disorders are common in prisoners. The transition between prison and community poses risks in terms of a disruption to treatment and care and a deterioration in health. Studies have demonstrated an increased risk of drug-related deaths among former prisoners during the first weeks following release from custody. The aim of this scoping review is to provide an up-to-date account of evidence from record linkage studies about drug-related deaths among former adult prisoners.

Methods and analysis

This is a protocol for a scoping review. The framework for conducting scoping reviews by Arksey and O'Malley, in addition to the methodology and guidance developed by the Joanna Briggs Institute (JBI) and the JBI Collaboration (JBIC) will be followed. MEDLINE, EMBASE, PsychINFO and Web of Science will be searched for studies from 2011 to 2021 using search terms relating to 'mortality', 'drugs' and 'ex-prisoner'. A preliminary search in MEDLINE identified at least 473 potentially eligible papers. Two authors will independently screen all titles and abstracts for eligibility using the inclusion and exclusion criteria. There will be no geographical restrictions but non-English language papers will be excluded. Data will be extracted using a piloted customised charting form and the findings will be mapped

For peer review only

(guided by the population, concept and context ('PCC') inclusion criteria) and grouped by main conceptual categories (including methodology, key findings and gaps in the research).

Discussion, ethics and dissemination

This scoping review will identify, map and summarise publicly available sources of research and therefore does not require ethical approval. The results will inform our empirical investigation of ex-prisoner mortality as well as being shared with key stakeholders, disseminated in a peer-reviewed journal and presented at relevant conferences.

Strengths and limitations of this study

- The proposed methodology for conducting our scoping review, including the use of the framework by Arksey and O'Malley, and methodology and guidance by the Joanna Briggs Institute is outlined by this protocol.
- The PRISMA-ScR (Preferred Reporting Items for Systematic reviews and Meta-Analyses extension for Scoping Reviews) checklist and guidance will be used to report the proposed review.
- MEDLINE, EMBASE, PsychINFO and Web of Science will be searched for studies from 2011 to 2021 using search terms relating to 'mortality', 'drugs' and 'ex-prisoner'.
- There will be no geographical restrictions but non-English language papers will be excluded.
- The proposed scoping review will map research evidence in this area, and may help identify and profile former prisoners who are most at risk, and inform potential interventions and future research and policy.

Introduction

The world prison population is estimated to be more than 11 million [1]. Prison population rates vary by regions; the world prison population rate is 145 per 100,000, however, the highest rate of 655 per 100,000 is found in the United States of America with over 2.1 million prisoners incarcerated [1]. Prisoners often have a complex mix of health and care needs including needs relating to mental and physical ill-health conditions, homelessness, unemployment and substance addictions [2]. Compared with the general population, prisoners have more physical and mental health problems and prison confinement serves as an opportunistic setting for the health care of this population [3, 4]. The provision of care to this population is expected to be equivalent to the care that is delivered to the wider community, and according to the United Nations (1990) *Basic Principles for the Treatment of Prisoners*, prisoners are entitled to access health services available in their resident country without discrimination [5]. However, the transition between prison and community including the transfer of care poses risks in terms of a disruption to treatment and care and a deterioration in health [4].

Mortality risk among former prisoners has been extensively studied using epidemiological methodology. Previous systematic reviews and meta-analyses among ex-prisoners have found an increased risk of death following release from prison, however, reviews have been limited by high levels of heterogeneity. A systematic review, published in 2012, explored the use of record linkage methods to examine deaths in ex-prisoners between 1998 and 2011, finding 29 publications (based

For peer review only

on 25 studies) [6]. The methodological review found limitations in both study quality and high levels of heterogeneity in the study design, analysis and findings of eligible publications [6]. Furthermore, most included studies were conducted in the United States of America, United Kingdom or Australia, therefore limiting the generalisability of the findings [6]. The review found an elevated risk of all-cause mortality, unnatural death, suicide and drug-related death among ex-prisoners for more than one year after release [6]. A separate systematic review and meta-analysis of publications between 1980 and 2011, explored the literature on studies of mortality in released prisoners using linkage of prisoner and mortality databases [7]. There is a high level of overlap in publications included in these systematic reviews [6, 7]. The review, of 18 cohorts, totalling more than 400,000 released prisoners and 26,163 deaths, was again limited by high heterogeneity of death rates as a result of differences in the study designs of primary publications [7]. The review found an increased risk of death from all causes, and in particular from suicide, homicide and drugs, with approximately one-fifth of deaths caused by drugs [7].

Substance use disorders are common in prisoners with approximately a third of male and a half of female prisoners entering prison with a drug use disorder [8]. Studies have demonstrated an increased risk of drug-related deaths among ex-prisoners following release from custody [9-11]. A meta-analysis of deaths among former prisoners (studies published between 1966 and 2009), including six studies (n=1,033 deaths), reported that 59% of deaths within the first three months of release were due to drug-related causes; this increased to 76% in the first two weeks after release [12]. The review focused on exploring mortality during the first 12 weeks following release from prison only and data were extracted from eligible publications for this period [12]. The meta-analysis reported a three to eight-fold increase in risk of drug-related death in the first two weeks after release from prison compared to the following 10 weeks (although the risk remained elevated for the first four weeks) [12]. However, in the meta-analysis, heterogeneity was present between countries (United States of America, United Kingdom or Australia) [12]. There is a high level of overlap in publications included in the meta-analysis and in the reviews by Kinner *et al.* and Zlodre and Fazel [6, 7, 12]. A scoping review between 2010 and 2020, found 43 publications relating to opioid overdose prevention in justice-involved populations (including correctional and community settings), of which 16 were cohort studies with data linkage [13]. The scoping review identified five core domains regarding opioid overdose prevention: acceptability, accessibility, effectiveness, feasibility and participant overdose risk [13]. The review found evidence of an immediate risk of overdose following release from prison; with the risk factors for opioid overdose including access to drugs, contact with drug using social groups and lack of social and socio-economic support [13]. Often, this at-risk group had contact with community-based service providers (after their release and before overdose) thereby pointing to opportunities for overdose prevention interventions during this period [13].

A recent systematic review found that interventions for prisoners with a mental health condition improved the transition process from prison to the community in terms of sustained contact with mental health services [14]. The interventions included in the review differed, for example, in stages of prison release and content, and further evidence is required [14]. Further research of at-risk groups on release from prison may help address their unmet needs, lead to more effective care, reduce negative outcomes and improve public health. This scoping review will focus specifically on studies that use record linkage methodology to investigate drug-related deaths in former prisoners as part of the work programme that is being undertaken by the Administrative Data Research Centre, Northern Ireland.

Purpose of review

For peer review only

The purpose of this scoping review is to perform an international review of the literature in record linkage studies of drug-related deaths among former adult prisoners. This review will add to current understanding by updating and mapping research evidence in this area, identify and profile former prisoners who are most at risk, and inform potential interventions and future research and policy.

Methods and analysis

This is a protocol for a scoping review. The framework for conducting scoping reviews by Arksey and O'Malley, in addition to the methodology and guidance developed by the Joanna Briggs Institute (JBI) and the JBI Collaboration (JBIC) will be followed to ensure rigour and transparency in this review [15, 16]. Our methodology will follow the first five stages of the framework as detailed below. As recommended by the JBI, population, concept and context ('PCC') will be incorporated into the scoping review title and research questions and the inclusion criteria will provide clear details of the PCC [16]. The PRISMA-ScR (Preferred Reporting Items for Systematic reviews and Meta-Analyses extension for Scoping Reviews) checklist and guidance will be used to report this review [17].

Stage 1: identifying the research question

The research questions are:

1. What is the scope of the literature on record linkage studies of drug-related deaths among former adult prisoners who have been released to the community?
2. How is research conducted on this topic?
3. What methodologies are used?
4. What are the findings in relation to mortality?
5. Where are the knowledge gaps on this topic?

Stage 2: identifying relevant studies

MEDLINE, EMBASE, PsychINFO and Web of Science will be searched for studies from 2011 to date of search using keywords and index headings (modified for searches in each database as required). The start date of 2011 was chosen for this scoping review to summarise the most recent evidence in this field of research. The search terms relate to one of the following three key areas: 'mortality', 'drugs' and 'ex-prisoner' and their variants. The search terms for the strategy were identified from previously published key literature and through discussion between JC and MD (with assistance from the Subject Librarian for the School of Medicine, Dentistry and Biomedical Sciences in Queen's University Belfast). A search strategy has been developed for MEDLINE by JC and MD with assistance from the Subject Librarian. This search strategy has been provided with this protocol as supplementary material (see Appendix 1). JC and MD will develop further search strategies for other databases to be searched. Final search strategies will be provided with the published review. All reference lists of included studies will be screened for the addition of any further publications. The search strategy will be limited to publications available in English due to resources for translation.

Stage 3: study selection

For peer review only - http://bmjopen.bmj.com/site/about/guidelines.xhtml

JC will perform all searches in the databases. The results across all of the databases will be combined in Mendeley Reference Manager and any duplicate publications will be removed. Two authors will independently screen all titles and abstracts for eligibility using the inclusion criteria. Any publications not eligible for inclusion will be excluded. If an abstract is not available or there is uncertainty over inclusion, the full publication will be screened. Full publication screening for inclusion will be performed independently by two authors. Any discrepancies between the two authors will be discussed with a third author. Authors of publications will be contacted for more information, if relevant.

Eligibility will be based on the pre-specified inclusion criteria in this protocol. The inclusion and exclusion criteria are as follows:

Population

The population will include adults (defined as 18 years and older) who have been imprisoned and released to the community. Individuals released from custodial placements such as young offender institutions will be excluded. Individuals remaining in prison custody (for example prisoners on remand and sentenced prisoners) will be excluded. There will be no exclusion on gender.

Concept

The key concepts revolve around record linkage of drug-related deaths in adults who have been imprisoned. Included studies must use data linkage (or similar meaning terms) to determine mortality outcomes following release from prison. Studies with no data linkage will be excluded. Only studies reporting cause-specific mortality (i.e. drug-related deaths) for either the entire study population or a subset of the study population will be included.

Context

All geographical locations will be included. The review will include research from peer-review journals. Qualitative studies, commentaries, editorials and conference abstracts will be excluded.

Stage 4: charting the data

Charting the data will allow extraction of the data from the eligible studies and a draft charting form has been developed to record key information (see Appendix 2). The development of the form has been informed by relevant previous literature, reviews and the content of RECORD (REporting of studies Conducted using Observational Routinely-collected health Data) statement [18]. This form has been piloted by JC and INO as part of the protocol development stage. The draft charting form may be refined by authors during the review, as required. The charting form will be tested by at least two authors to ensure that all relevant key information is extracted consistently and accurately. Two authors will independently extract information from the eligible publications using this form. Any discrepancies between the two authors will be discussed with a third author. Authors of publications will be contacted for more information, if necessary.

Stage 5: collating, summarising and reporting the results

For peer review only

The data will be presented in relation to the purpose of the review (an international review of record linkage studies of drug-related deaths among former adult prisoners) and in the format that will answer the scoping review questions. The results will be presented as a map of the data (guided by the PCC inclusion criteria). The extracted data will be summarised and presented in tables. The results will also be organised according to the main conceptual categories (including methodology, key findings and gaps in the research).

Patient and public involvement

Dr Richard Kirk is a core member of this review team. Dr Kirk is the Clinical Director of Healthcare in Prison in Northern Ireland. No additional patient and public involvement.

Discussion, ethics and dissemination

This review will identify, map and summarise publicly available sources of research and therefore does not require ethical approval. This work will review the evidence in record linkage studies of drug-related deaths among former adult prisoners and may be used to identify those most at risk, and inform future research and policy. The results of the review will inform our empirical investigation of ex-prisoner mortality as well as being shared with key stakeholders, disseminated in a peer-reviewed journal and presented at relevant conferences.

Contributions

JC, DO'R and MD conceived the scoping review idea. JC and MD developed the scoping review protocol, scoping review title, research questions and methods. JC drafted the manuscript. MD edited the first draft of the manuscript. JC and INO piloted the draft charting form as part of this protocol development. All co-authors reviewed the manuscript and have given final approval for publication.

Funding

This work is funded by a grant awarded to the ADRC NI by the Economic and Social Research Council (ESRC) (grant number ES/S00744X/1).

Competing interests

The authors declare that they have no competing interests.

Acknowledgements

The authors would like to acknowledge Richard Fallis, Subject Librarian for the School of Medicine, Dentistry and Biomedical Sciences in Queen's University Belfast, for assisting in the development of the search terms and search strategy to be used in this scoping review.

For peer review only

References

1. Walmsley R. World Prison Population List. Twelfth edition. World Prison Brief. Institute for Criminal Policy Research 2018.
2. Rutherford M, Duggan S. Meeting complex health needs in prisons. Public health 2009;123:415-8.
3. Kinner SA, Streitberg L, Butler T, Levy M. Prisoner and ex-prisoner health - improving access to primary care. Aust Fam Physician 2012;41(7):535-7.
4. Bradshaw R, Pordes BA, Trippier H, et al; Guideline Committee for the NICE guideline on physical health of people in prisons and the NICE guideline on mental health of adults in contact with the criminal justice system. The health of prisoners: summary of NICE guidance. *BMJ* 2017;356:j1378. doi: 10.1136/bmj.j1378. PMID: 28348003.
5. Gatherer A, Jürgens R, Stöver H. Health in prisons: a WHO guide to the essentials in prison health. WHO Regional Office Europe; 2007.
6. Kinner SA, Forsyth S, Williams G. Systematic review of record linkage studies of mortality in ex-prisoners: why (good) methods matter. *Addiction* 2013;108(1):38-49.
7. Zlodre J, Fazel S. All-cause and external mortality in released prisoners: systematic review and meta-analysis. Am J Public Health 2012;102(12):e67-75.
8. Fazel S, Yoon IA, Hayes AJ. Substance use disorders in prisoners: an updated systematic review and meta-regression analysis in recently incarcerated men and women. *Addiction* 2017;112(10):1725-1739.
9. Bird SM, Fischbacher CM, Graham L, Fraser A. Impact of opioid substitution therapy for Scotland's prisoners on drug-related deaths soon after prisoner release. *Addiction* 2015;110(10):1617-24.
10. Bukten A, Stavseth MR, Skurtveit S, et al. High risk of overdose death following release from prison: variations in mortality during a 15-year observation period. *Addiction* 2017;112(8):1432-1439.
11. Alex B, Weiss DB, Kaba F, et al. Death After Jail Release. *J Correct Health Care* 2017;23(1):83-87.
12. Merrall EL, Kariminia A, Binswanger IA,et al. Meta-analysis of drug-related deaths soon after release from prison. Addiction 2010;105(9):1545-54.
13. Grella CE, Ostlie E, Scott CK,et al. A scoping review of factors that influence opioid overdose prevention for justice-involved populations. *Subst Abuse Treat Prev Policy* 2021;16(1):19. doi: 10.1186/s13011-021-00346-1.
14. Hopkin G, Evans-Lacko S, Forrester A,et al. Interventions at the Transition from Prison to the Community for Prisoners with Mental Illness: A Systematic Review. *Adm Policy Ment Health*. 2018;45(4):623-634.
15. Arksey H, O'Malley L. Scoping studies: towards a methodological framework. *Int J Soc Res Methodol* 2015; 8:1:19-32
16. Peters MDJ, Godfrey C, McInerney P, et al. Chapter 11: Scoping Reviews (2020 version). In: Aromataris E, Munn Z (Editors). JBI Manual for Evidence Synthesis, JBI, 2020. Available from https://synthesismanual.jbi.global. https://doi.org/10.46658/JBIMES-20-12
17. Tricco AC, Lillie E, Zarin W, et al. PRISMA extension for scoping reviews (PRISMA-ScR): checklist and explanation. *Ann Intern Med* 2018;169(7):467-473.
18. Benchimol EI, Smeeth L, Guttmann A, et al. RECORD Working Committee. The REporting of studies Conducted using Observational Routinely-collected health Data (RECORD) statement. *PLoS Med* 2015 Oct 6;12(10):e1001885. doi: 10.1371/journal.pmed.1001885. PMID: 26440803; PMCID: PMC4595218.

For peer review only

Record linkage studies of drug-related deaths among former adult prisoners who have been released to the community: a scoping review protocol

Cooper JA [1,2]*, Onyeka IN [1,2], O'Reilly D [1,2], Kirk R [3], Donnelly M [1,2]

[1] Centre for Public Health, Queen's University Belfast, Royal Hospitals Site, Grosvenor Road, Belfast, UK

[2] Administrative Data Research Centre Northern Ireland (ADRC NI), Centre for Public Health, Queen's University Belfast, Royal Hospitals Site, Grosvenor Road, Belfast, UK

[3] South Eastern Health and Social Care Trust, Ulster Hospital, Dundonald, UK

*corresponding author

Supplementary material

Appendix 1

Ovid MEDLINE(R) ALL <1946 to August 17, 2021>

1 Prisoners/ 17290

2 ex-prisoner*.mp. 201

3 former prisoner*.mp. 262

4 inmate*.mp. 5441

5 ex-inmate*.mp. 17

6 former inmate*.mp. 102

7 Criminals/ 5440

8 ex-criminal*.mp. [mp=title, abstract, original title, name of substance word, subject heading word, floating sub-heading word, keyword heading word, organism supplementary concept word, protocol supplementary concept word, rare disease supplementary concept word, unique identifier, synonyms] 0

9 former criminal*.mp. 0

10 convict*.mp. 7081

11 ex-convict*.mp. 12

12 former convict*.mp. 3

13 detainee*.mp. 989

14 ex-detainee*.mp. 8

15 former detainee*.mp. 8

16 Prisons/ 10227

For peer review only

17 Jails/ 66

18 gaol*.mp. 150

19 imprison*.mp. 2645

20 incarcerat*.mp. [mp=title, abstract, original title, name of substance word, subject heading word, floating sub-heading word, keyword heading word, organism supplementary concept word, protocol supplementary concept word, rare disease supplementary concept word, unique identifier, synonyms] 12802

21 detention*.mp. 3423

22 correction*.mp.235912

23 confinement.mp. 22002

24 Correctional Facilities/ 69

25 penal institution*.mp. 133

26 penitentiar*.mp. [mp=title, abstract, original title, name of substance word, subject heading word, floating sub-heading word, keyword heading word, organism supplementary concept word, protocol supplementary concept word, rare disease supplementary concept word, unique identifier, synonyms] 693

27 remand.mp. 295

28 offender*.mp. 12056

29 ex-offender*.mp. 102

30 former offender*.mp. 4

31 after release.mp. 4226

32 prison release.mp. 136

33 released prisoner*.mp. 134

34 following release.mp. 1117

35 recently released.mp. 1911

36 newly released.mp. 786

37 postrelease.mp. 341

38 post-release.mp. 787

39 liberat*.mp. 38930

40 "Cause of Death"/ or Death/ 69103

41 Mortality/ 47259

42 Fatal Outcome/ 65463

43 fatal*.mp. 214521

For peer review only

44 lethal*.mp. 163790

45 Substance-Related Disorders/ 99086

46 Opioid-Related Disorders/ 17378

47 drug abuse*.mp. 21345

48 drug dependen*.mp. 6424

49 Drug Misuse/ or Prescription Drug Misuse/ or Substance Abuse, Intravenous/ 18137

50 Drug Overdose/ 12514

51 drug poisoning.mp. 705

52 Drug Users/ or Injections, Intravenous/ 85588

53 substance abuse*.mp. 55910

54 substance dependen*.mp. 3072

55 substance misuse*.mp. 3022

56 substance use*.mp. 42442

57 Illicit Drugs/ 11763

58 substance poisoning.mp. 17

59 street drug*.mp. 735

60 toxicity.mp. 750642

61 Opiate Overdose/ or Analgesics, Opioid/ 49884

62 Heroin Dependence/ or Heroin/ 13437

63 drug withdrawal.mp. 3932

64 accidental poisoning.mp. 912

65 Benzodiazepines/ 22377

66 Cocaine-Related Disorders/ or Cocaine/ or Crack Cocaine/ or Cocaine Smoking/ 30584

67 Narcotics/ 16548

68 1 or 2 or 3 or 4 or 5 or 6 or 7 or 8 or 9 or 10 or 11 or 12 or 13 or 14 or 15 or 16 or 17 or 18 or 19 or 20 or 21 or 22 or 23 or 24 or 25 or 26 or 27 or 28 or 29 or 30 or 31 or 32 or 33 or 34 or 35 or 36 or 37 or 38 or 39 353126

69 40 or 41 or 42 or 43 or 44 475646

70 45 or 46 or 47 or 48 or 49 or 50 or 51 or 52 or 53 or 54 or 55 or 56 or 57 or 58 or 59 or 60 or 61 or 62 or 63 or 64 or 65 or 66 or 67 1110035

71 68 and 69 and 70 530

72 limit 71 to english language 473

For peer review only

For peer review only

Record linkage studies of drug-related deaths among former adult prisoners who have been released to the community: a scoping review protocol

Cooper JA [1,2]*, Onyeka IN [1,2], O'Reilly D [1,2], Kirk R [3], Donnelly M [1,2]

[1] Centre for Public Health, Queen's University Belfast, Royal Hospitals Site, Grosvenor Road, Belfast, UK

[2] Administrative Data Research Centre Northern Ireland (ADRC NI), Centre for Public Health, Queen's University Belfast, Royal Hospitals Site, Grosvenor Road, Belfast, UK

[3] South Eastern Health and Social Care Trust, Ulster Hospital, Dundonald, UK

*corresponding author

For peer review only

Supplementary material

Appendix 2

DATA CHARTING FORM

**Study assessor initials**		
**Date of completion of data charting form**		
**Item**	***Notes on item***	**Data extracted**
**Study reference number**		
**Study assessment number**		
**Lead author**		
**Title**		

**Year of publication**		
**Origin, country of origin**		
**Journal name**		
**Study design**		
**Citation**		
**Setting**	*Stated setting*	
	Stated locations	
	Stated relevant dates	
**Participants**	*Stated study population*	
	Stated type of prison	
	Stated age inclusions (or any stated age exclusions)	
	Stated methods of study population selection (type of prisoner data records)	
	Stated methods of follow-up (for example, record linkage or interview)	
	Stated rationale for the choice of cases and controls (case-control study only)	
	Stated matching criteria (case-control study only)	

For peer review only

	Stated number of controls per case (case-control study only)	
**Data sources**	*Stated sources of data*	
	Stated sources of comparator data	
**Bias**	*Stated bias*	
	Stated efforts to address potential sources of bias	
	Stated methods for dealing with repeated incarcerations?	
**Study size**	*Stated study size*	
**Statistical methods**	*Stated statistical methods*	
	Stated methods controlling for confounders and methods for examining subgroups and interactions	
	Stated sensitivity analyses	
	Stated time period examined after prison release	
	Stated how missing data was addressed	
	Stated how loss to follow-up was addressed	

For peer review only

	Stated how matching of case and controls was addressed (case-control study only)	
**Linkage**	*Stated linkage i.e. if person-level, institution-level or other data linkage across two or more databases*	
	Stated types of linked databases	
	Stated methods of linkage	
**Main outcomes**	*Stated outcome events or summary measures*	
**Main results**	*Stated unadjusted estimate and, if applicable, confounder adjusted estimates, their precision, which confounders were adjusted for.*<br><br>*Reported for all cause and drug-related deaths*	
	Stated age at release (mean/median/SD/range)	
	Stated length of incarceration (mean/median/SD/range)	
**Limitations**	*Stated limitations of study including sources of potential bias or imprecision (are the results generalisable)*	
**Quality assessment checklist or technique**	*Stated if quality assessment checklist or technique was used*	

For peer review only

BMJ Open

Record linkage studies of drug-related deaths among former adult prisoners who have been released to the community: a scoping review protocol

Journal:	*BMJ Open*
Manuscript ID	bmjopen-2021-056598.R1
Article Type:	Protocol
Date Submitted by the Author:	31-Jan-2022
Complete List of Authors:	Cooper, Janine; Queen's University Belfast, Centre for Public Health, Queen's University Belfast, Royal Hospitals Site, Grosvenor Road, Belfast, UK; Queen's University Belfast, Administrative Data Research Centre Northern Ireland (ADRC NI), Centre for Public Health, Queen's University Belfast, Royal Hospitals Site, Grosvenor Road, Belfast, UK<br>Onyeka, Ifeoma ; Queen's University Belfast, Centre for Public Health, Queen's University Belfast, Royal Hospitals Site, Grosvenor Road, Belfast, UK; Queen's University Belfast, Administrative Data Research Centre Northern Ireland (ADRC NI), Centre for Public Health, Queen's University Belfast, Royal Hospitals Site, Grosvenor Road, Belfast, UK<br>OReilly, Dermot; Queen's University Belfast, Centre for Public Health, Queen's University Belfast, Royal Hospitals Site, Grosvenor Road, Belfast, UK; Queen's University Belfast, Administrative Data Research Centre Northern Ireland (ADRC NI), Centre for Public Health, Queen's University Belfast, Royal Hospitals Site, Grosvenor Road, Belfast, UK<br>Kirk, Richard; South Eastern Health and Social Care Trust, South Eastern Health and Social Care Trust, Ulster Hospital, Dundonald, UK<br>Donnelly, Michael; Queen's University Belfast, Centre for Public Health, Queen's University Belfast, Royal Hospitals Site, Grosvenor Road, Belfast, UK; Queen's University Belfast, Administrative Data Research Centre Northern Ireland (ADRC NI), Centre for Public Health, Queen's University Belfast, Royal Hospitals Site, Grosvenor Road, Belfast, UK
**Primary Subject Heading**:	Epidemiology
Secondary Subject Heading:	Epidemiology, Mental health, Public health
Keywords:	EPIDEMIOLOGY, MENTAL HEALTH, PUBLIC HEALTH

SCHOLARONE™
Manuscripts

For peer review only - http://bmjopen.bmj.com/site/about/guidelines.xhtml

Record linkage studies of drug-related deaths among former adult prisoners who have been released to the community: a scoping review protocol

Cooper JA [1,2]*, Onyeka IN [1,2], O'Reilly D [1,2], Kirk R [3], Donnelly M [1,2]

[1] Centre for Public Health, Queen's University Belfast, Royal Hospitals Site, Grosvenor Road, Belfast, UK

[2] Administrative Data Research Centre Northern Ireland (ADRC NI), Centre for Public Health, Queen's University Belfast, Royal Hospitals Site, Grosvenor Road, Belfast, UK

[3] South Eastern Health and Social Care Trust, Ulster Hospital, Dundonald, UK

*corresponding author

Corresponding author contact: Cooper JA, Centre for Public Health, Queen's University Belfast, Royal Hospitals Site, Grosvenor Road, Belfast, UK. Administrative Data Research Centre Northern Ireland (ADRC NI), Centre for Public Health, Queen's University Belfast, Royal Hospitals Site, Grosvenor Road, Belfast, UK. Email: j.cooper@qub.ac.uk

For peer review only

Abstract

Introduction

Prisoners often have a complex mix of health and care needs including needs relating to mental and physical ill-health conditions. In addition, substance use disorders are common in prisoners. The transition between prison and community poses risks in terms of a disruption to treatment and care and a deterioration in health. Studies have demonstrated an increased risk of drug-related deaths among former prisoners during the first weeks following release from custody. The aim of this scoping review is to provide an up-to-date account of evidence from record linkage studies about drug-related deaths among former adult prisoners.

Methods and analysis

This is a protocol for a scoping review. The framework for conducting scoping reviews by Arksey and O'Malley, in addition to the methodology and guidance developed by the Joanna Briggs Institute (JBI) and the JBI Collaboration (JBIC) will be followed. MEDLINE, EMBASE, PsychINFO and Web of Science will be searched for studies from 2011 to 2021 using search terms relating to 'mortality', 'drugs' and 'ex-prisoner'. A preliminary search in MEDLINE identified at least 473 potentially eligible papers. Two authors will independently screen all titles and abstracts for eligibility using the inclusion and exclusion criteria. There will be no geographical restrictions but non-English language papers will be excluded. Data will be extracted using a piloted customised charting form and the findings will be mapped

(guided by the population, concept and context ('PCC') inclusion criteria) and grouped by main conceptual categories (including methodology, key findings and gaps in the research).

Discussion, ethics and dissemination

This scoping review will identify, map and summarise publicly available sources of research and therefore does not require ethical approval. The results will inform our empirical investigation of ex-prisoner mortality as well as being shared with key stakeholders, disseminated in a peer-reviewed journal and presented at relevant conferences.

Strengths and limitations of this study

- The proposed methodology for conducting our scoping review, including the use of the framework by Arksey and O'Malley, and methodology and guidance by the Joanna Briggs Institute is outlined by this protocol.
- The PRISMA-ScR (Preferred Reporting Items for Systematic reviews and Meta-Analyses extension for Scoping Reviews) checklist and guidance will be used to report the proposed review.
- MEDLINE, EMBASE, PsychINFO and Web of Science will be searched for studies from 2011 to 2021 using search terms relating to 'mortality', 'drugs' and 'ex-prisoner'.
- There will be no geographical restrictions but non-English language papers will be excluded.
- The proposed scoping review will map research evidence in this area, and may help identify and profile former prisoners who are most at risk, and inform potential interventions and future research and policy.

Introduction

The world prison population is estimated to be more than 11 million [1]. Prison population rates vary by regions; the world prison population rate is 145 per 100,000, however, the highest rate of 655 per 100,000 is found in the United States of America with over 2.1 million prisoners incarcerated [1]. Prisoners often have a complex mix of health and care needs including needs relating to mental and physical ill-health conditions, homelessness, unemployment and substance addictions [2]. Compared with the general population, prisoners have more physical and mental health problems and prison confinement serves as an opportunistic setting for the health care of this population [3, 4]. The provision of care to this population is expected to be equivalent to the care that is delivered to the wider community, and according to the United Nations (1990) *Basic Principles for the Treatment of Prisoners*, prisoners are entitled to access health services available in their resident country without discrimination [5]. However, the transition between prison and community including the transfer of care poses risks in terms of a disruption to treatment and care and a deterioration in health [4].

Mortality risk among former prisoners has been extensively studied using epidemiological methodology. Previous systematic reviews and meta-analyses among ex-prisoners have found an increased risk of death following release from prison, however, reviews have been limited by high levels of heterogeneity. A systematic review, published in 2012, explored the use of record linkage methods to examine deaths in ex-prisoners between 1998 and 2011, finding 29 publications (based

For peer review only

on 25 studies) [6]. The methodological review found limitations in both study quality and high levels of heterogeneity in the study design, analysis and findings of eligible publications [6]. Furthermore, most included studies were conducted in the United States of America, United Kingdom or Australia, therefore limiting the generalisability of the findings [6]. The review found an elevated risk of all-cause mortality, unnatural death, suicide and drug-related death among ex-prisoners for more than one year after release [6]. A separate systematic review and meta-analysis of publications between 1980 and 2011, explored the literature on studies of mortality in released prisoners using linkage of prisoner and mortality databases [7]. There is a high level of overlap in publications included in these systematic reviews [6, 7]. The review, of 18 cohorts, totalling more than 400,000 released prisoners and 26,163 deaths, was again limited by high heterogeneity of death rates as a result of differences in the study designs of primary publications [7]. The review found an increased risk of death from all causes, and in particular from suicide, homicide and drugs, with approximately one-fifth of deaths caused by drugs [7].

Substance use disorders are common in prisoners with approximately a third of male and a half of female prisoners entering prison with a drug use disorder [8]. Studies have demonstrated an increased risk of drug-related deaths among ex-prisoners following release from custody [9-11]. A meta-analysis of deaths among former prisoners (studies published between 1966 and 2009), including six studies (n=1,033 deaths), reported that 59% of deaths within the first three months of release were due to drug-related causes; this increased to 76% in the first two weeks after release [12]. The review focused on exploring mortality during the first 12 weeks following release from prison only and data were extracted from eligible publications for this period [12]. The meta-analysis reported a three to eight-fold increase in risk of drug-related death in the first two weeks after release from prison compared to the following 10 weeks (although the risk remained elevated for the first four weeks) [12]. However, in the meta-analysis, heterogeneity was present between countries (United States of America, United Kingdom or Australia) [12]. There is a high level of overlap in publications included in the meta-analysis and in the reviews by Kinner *et al.* and Zlodre and Fazel [6, 7, 12]. A scoping review between 2010 and 2020, found 43 publications relating to opioid overdose prevention in justice-involved populations (including correctional and community settings), of which 16 were cohort studies with data linkage [13]. The scoping review identified five core domains regarding opioid overdose prevention: acceptability, accessibility, effectiveness, feasibility and participant overdose risk [13]. The review found evidence of an immediate risk of overdose following release from prison; with the risk factors for opioid overdose including access to drugs, contact with drug using social groups and lack of social and socio-economic support [13]. Often, this at-risk group had contact with community-based service providers (after their release and before overdose) thereby pointing to opportunities for overdose prevention interventions during this period [13].

A recent systematic review found that interventions for prisoners with a mental health condition improved the transition process from prison to the community in terms of sustained contact with mental health services [14]. The interventions included in the review differed, for example, in stages of prison release and content, and further evidence is required [14]. Further research of at-risk groups on release from prison may help address their unmet needs, lead to more effective care, reduce negative outcomes and improve public health. This scoping review will focus specifically on studies that use record linkage methodology to investigate drug-related deaths in former prisoners as part of the work programme that is being undertaken by the Administrative Data Research Centre, Northern Ireland.

Purpose of review

For peer review only

The purpose of this scoping review is to perform an international review of the literature in record linkage studies of drug-related deaths among former adult prisoners. This review will add to current understanding by updating and mapping research evidence in this area, identify and profile former prisoners who are most at risk, and inform potential interventions and future research and policy.

Methods and analysis

This is a protocol for a scoping review. The framework for conducting scoping reviews by Arksey and O'Malley, in addition to the methodology and guidance developed by the Joanna Briggs Institute (JBI) and the JBI Collaboration (JBIC) will be followed to ensure rigour and transparency in this review [15, 16]. Our methodology will follow the first five stages of the framework as detailed below. As recommended by the JBI, population, concept and context ('PCC') will be incorporated into the scoping review title and research questions and the inclusion criteria will provide clear details of the PCC [16]. The PRISMA-ScR (Preferred Reporting Items for Systematic reviews and Meta-Analyses extension for Scoping Reviews) checklist and guidance will be used to report this review [17].

Stage 1: identifying the research question

The research questions are:

1. What is the scope of the literature on record linkage studies of drug-related deaths among former adult prisoners who have been released to the community?
2. How is research conducted on this topic?
3. What methodologies are used?
4. What are the findings in relation to mortality?
5. Where are the knowledge gaps on this topic?

Stage 2: identifying relevant studies

MEDLINE, EMBASE, PsychINFO and Web of Science will be searched for studies from January 2011 to September 2021 using keywords and index headings (modified for searches in each database as required). The start date of 2011 was chosen for this scoping review to summarise the most recent evidence in this field of research. The search terms relate to one of the following three key areas: 'mortality', 'drugs' and 'ex-prisoner' and their variants. The search terms for the strategy were identified from previously published key literature and through discussion between JC and MD (with assistance from the Subject Librarian for the School of Medicine, Dentistry and Biomedical Sciences in Queen's University Belfast). A search strategy has been developed for MEDLINE by JC and MD with assistance from the Subject Librarian. This search strategy has been provided with this protocol as supplementary material (see Appendix 1). JC and MD will develop further search strategies for other databases to be searched. Final search strategies will be provided with the published review. All reference lists of included studies will be screened for the addition of any further publications. The search strategy will be limited to publications available in English due to resources for translation.

Stage 3: study selection

For peer review only - http://bmjopen.bmj.com/site/about/guidelines.xhtml

JC will perform all searches in the databases. The results across all of the databases will be combined in Mendeley Reference Manager and any duplicate publications will be removed. Two authors will independently screen all titles and abstracts for eligibility using the inclusion criteria. Any publications not eligible for inclusion will be excluded. If an abstract is not available or there is uncertainty over inclusion, the full publication will be screened. Full publication screening for inclusion will be performed independently by two authors. Any discrepancies between the two authors will be discussed with a third author. Authors of publications will be contacted for more information, if relevant.

Eligibility will be based on the pre-specified inclusion criteria in this protocol. The inclusion and exclusion criteria are as follows:

Population

The population will include adults (defined as 18 years and older) who have been imprisoned and released to the community. Individuals released from custodial placements such as young offender institutions will be excluded. Individuals remaining in prison custody (for example prisoners on remand and sentenced prisoners) will be excluded. There will be no exclusion on gender.

Concept

The key concepts revolve around record linkage of drug-related deaths in adults who have been imprisoned. Included studies must use data linkage (or similar meaning terms) to determine mortality outcomes following release from prison. Studies with no data linkage will be excluded. Only studies reporting cause-specific mortality (i.e. drug-related deaths) for either the entire study population or a subset of the study population will be included.

Context

All geographical locations will be included. The review will include research from peer-review journals. Qualitative studies, commentaries, editorials and conference abstracts will be excluded.

Stage 4: charting the data

Charting the data will allow extraction of the data from the eligible studies and a draft charting form has been developed to record key information (see Appendix 2). The development of the form has been informed by relevant previous literature, reviews and the content of RECORD (REporting of studies Conducted using Observational Routinely-collected health Data) statement [18]. This form has been piloted by JC and INO as part of the protocol development stage. The draft charting form may be refined by authors during the review, as required. The charting form will be tested by at least two authors to ensure that all relevant key information is extracted consistently and accurately. Two authors will independently extract information from the eligible publications using this form. Any discrepancies between the two authors will be discussed with a third author. Authors of publications will be contacted for more information, if necessary.

Stage 5: collating, summarising and reporting the results

For peer review only

The data will be presented in relation to the purpose of the review (an international review of record linkage studies of drug-related deaths among former adult prisoners) and in the format that will answer the scoping review questions. We will analyse and summarise reports of included studies that used data linkage to determine mortality outcomes following release from prison in terms of, for example, crude mortality rates and standardised mortality ratios. The results will be presented as a map of the data (guided by the PCC inclusion criteria). The extracted data will be summarised and presented in tables. The results will also be organised according to the main conceptual categories (including methodology, key findings and gaps in the research).

Patient and public involvement

Dr Richard Kirk is a core member of this review team. Dr Kirk is the Clinical Director of Healthcare in Prison in Northern Ireland. No additional patient and public involvement.

Discussion, ethics and dissemination

This review will identify, map and summarise publicly available sources of research and therefore does not require ethical approval. This work will review the evidence in record linkage studies of drug-related deaths among former adult prisoners and may be used to identify those most at risk, and inform future research and policy. Where possible, time period examined after prison release and information on specific drugs will be reported in relation to drug-related mortality. There are some limitations in this planned scoping review. The search strategy is limited to publications available in English due to resources for translation and the review does not include a search of the grey literature which may limit the interpretation of the findings. The results of the review will inform our empirical investigation of ex-prisoner mortality as well as being shared with key stakeholders, disseminated in a peer-reviewed journal and presented at relevant conferences.

Contributions

JC, DO'R, RK and MD conceived the scoping review idea. JC and MD developed the scoping review protocol, scoping review title, research questions and methods. JC drafted the manuscript. MD edited the first draft of the manuscript. JC and INO piloted the draft charting form as part of this protocol development. All co-authors reviewed the manuscript and have given final approval for publication.

Funding

This work is funded by a grant awarded to the ADRC NI by the Economic and Social Research Council (ESRC) (grant number ES/S00744X/1).

Competing interests

The authors declare that they have no competing interests.

For peer review only - http://bmjopen.bmj.com/site/about/guidelines.xhtml

Acknowledgements

The authors would like to acknowledge Richard Fallis, Subject Librarian for the School of Medicine, Dentistry and Biomedical Sciences in Queen's University Belfast, for assisting in the development of the search terms and search strategy to be used in this scoping review.

References

1. Walmsley R. World Prison Population List. Twelfth edition. World Prison Brief. Institute for Criminal Policy Research 2018.
2. Rutherford M, Duggan S. Meeting complex health needs in prisons. Public health 2009;123:415-8.
3. Kinner SA, Streitberg L, Butler T, Levy M. Prisoner and ex-prisoner health - improving access to primary care. Aust Fam Physician 2012;41(7):535-7.
4. Bradshaw R, Pordes BA, Trippier H, et al; Guideline Committee for the NICE guideline on physical health of people in prisons and the NICE guideline on mental health of adults in contact with the criminal justice system. The health of prisoners: summary of NICE guidance. *BMJ* 2017;356:j1378. doi: 10.1136/bmj.j1378. PMID: 28348003.
5. Gatherer A, Jürgens R, Stöver H. Health in prisons: a WHO guide to the essentials in prison health. WHO Regional Office Europe; 2007.
6. Kinner SA, Forsyth S, Williams G. Systematic review of record linkage studies of mortality in ex-prisoners: why (good) methods matter. *Addiction* 2013;108(1):38-49.
7. Zlodre J, Fazel S. All-cause and external mortality in released prisoners: systematic review and meta-analysis. Am J Public Health 2012;102(12):e67-75.
8. Fazel S, Yoon IA, Hayes AJ. Substance use disorders in prisoners: an updated systematic review and meta-regression analysis in recently incarcerated men and women. *Addiction* 2017;112(10):1725-1739.
9. Bird SM, Fischbacher CM, Graham L, Fraser A. Impact of opioid substitution therapy for Scotland's prisoners on drug-related deaths soon after prisoner release. *Addiction* 2015;110(10):1617-24.
10. Bukten A, Stavseth MR, Skurtveit S, et al. High risk of overdose death following release from prison: variations in mortality during a 15-year observation period. *Addiction* 2017;112(8):1432-1439.
11. Alex B, Weiss DB, Kaba F, et al. Death After Jail Release. *J Correct Health Care* 2017;23(1):83-87.
12. Merrall EL, Kariminia A, Binswanger IA,et al. Meta-analysis of drug-related deaths soon after release from prison. Addiction 2010;105(9):1545-54.
13. Grella CE, Ostlie E, Scott CK,et al. A scoping review of factors that influence opioid overdose prevention for justice-involved populations. *Subst Abuse Treat Prev Policy* 2021;16(1):19. doi: 10.1186/s13011-021-00346-1.
14. Hopkin G, Evans-Lacko S, Forrester A,et al. Interventions at the Transition from Prison to the Community for Prisoners with Mental Illness: A Systematic Review. *Adm Policy Ment Health*. 2018;45(4):623-634.
15. Arksey H, O'Malley L. Scoping studies: towards a methodological framework. *Int J Soc Res Methodol* 2015; 8:1:19-32
16. Peters MDJ, Godfrey C, McInerney P, et al. Chapter 11: Scoping Reviews (2020 version). In: Aromataris E, Munn Z (Editors). JBI Manual for Evidence Synthesis, JBI, 2020. Available from https://synthesismanual.jbi.global. https://doi.org/10.46658/JBIMES-20-12

17. Tricco AC, Lillie E, Zarin W, et al. PRISMA extension for scoping reviews (PRISMA-ScR): checklist and explanation. *Ann Intern Med* 2018;169(7):467-473.
18. Benchimol EI, Smeeth L, Guttmann A, et al. RECORD Working Committee. The REporting of studies Conducted using Observational Routinely-collected health Data (RECORD) statement. *PLoS Med* 2015 Oct 6;12(10):e1001885. doi: 10.1371/journal.pmed.1001885. PMID: 26440803; PMCID: PMC4595218.

For peer review only

Record linkage studies of drug-related deaths among former adult prisoners who have been released to the community: a scoping review protocol

Cooper JA [1,2]*, Onyeka IN [1,2], O'Reilly D [1,2], Kirk R [3], Donnelly M [1,2]

[1] Centre for Public Health, Queen's University Belfast, Royal Hospitals Site, Grosvenor Road, Belfast, UK

[2] Administrative Data Research Centre Northern Ireland (ADRC NI), Centre for Public Health, Queen's University Belfast, Royal Hospitals Site, Grosvenor Road, Belfast, UK

[3] South Eastern Health and Social Care Trust, Ulster Hospital, Dundonald, UK

*corresponding author

Supplementary material

Appendix 1

Ovid MEDLINE(R) ALL <1946 to August 17, 2021>

1 Prisoners/ 17290

2 ex-prisoner*.mp. 201

3 former prisoner*.mp. 262

4 inmate*.mp. 5441

5 ex-inmate*.mp. 17

6 former inmate*.mp. 102

7 Criminals/ 5440

8 ex-criminal*.mp. [mp=title, abstract, original title, name of substance word, subject heading word, floating sub-heading word, keyword heading word, organism supplementary concept word, protocol supplementary concept word, rare disease supplementary concept word, unique identifier, synonyms] 0

9 former criminal*.mp. 0

10 convict*.mp. 7081

11 ex-convict*.mp. 12

12 former convict*.mp. 3

13 detainee*.mp. 989

14 ex-detainee*.mp. 8

15 former detainee*.mp. 8

16 Prisons/ 10227

For peer review only

17 Jails/ 66

18 gaol*.mp. 150

19 imprison*.mp. 2645

20 incarcerat*.mp. [mp=title, abstract, original title, name of substance word, subject heading word, floating sub-heading word, keyword heading word, organism supplementary concept word, protocol supplementary concept word, rare disease supplementary concept word, unique identifier, synonyms] 12802

21 detention*.mp. 3423

22 correction*.mp.235912

23 confinement.mp. 22002

24 Correctional Facilities/ 69

25 penal institution*.mp. 133

26 penitentiar*.mp. [mp=title, abstract, original title, name of substance word, subject heading word, floating sub-heading word, keyword heading word, organism supplementary concept word, protocol supplementary concept word, rare disease supplementary concept word, unique identifier, synonyms] 693

27 remand.mp. 295

28 offender*.mp. 12056

29 ex-offender*.mp. 102

30 former offender*.mp. 4

31 after release.mp. 4226

32 prison release.mp. 136

33 released prisoner*.mp. 134

34 following release.mp. 1117

35 recently released.mp. 1911

36 newly released.mp. 786

37 postrelease.mp. 341

38 post-release.mp. 787

39 liberat*.mp. 38930

40 "Cause of Death"/ or Death/ 69103

41 Mortality/ 47259

42 Fatal Outcome/ 65463

43 fatal*.mp. 214521

For peer review only

44 lethal*.mp. 163790

45 Substance-Related Disorders/ 99086

46 Opioid-Related Disorders/ 17378

47 drug abuse*.mp. 21345

48 drug dependen*.mp. 6424

49 Drug Misuse/ or Prescription Drug Misuse/ or Substance Abuse, Intravenous/ 18137

50 Drug Overdose/12514

51 drug poisoning.mp. 705

52 Drug Users/ or Injections, Intravenous/ 85588

53 substance abuse*.mp. 55910

54 substance dependen*.mp. 3072

55 substance misuse*.mp. 3022

56 substance use*.mp. 42442

57 Illicit Drugs/ 11763

58 substance poisoning.mp. 17

59 street drug*.mp. 735

60 toxicity.mp. 750642

61 Opiate Overdose/ or Analgesics, Opioid/49884

62 Heroin Dependence/ or Heroin/13437

63 drug withdrawal.mp. 3932

64 accidental poisoning.mp. 912

65 Benzodiazepines/ 22377

66 Cocaine-Related Disorders/ or Cocaine/ or Crack Cocaine/ or Cocaine Smoking/ 30584

67 Narcotics/ 16548

68 1 or 2 or 3 or 4 or 5 or 6 or 7 or 8 or 9 or 10 or 11 or 12 or 13 or 14 or 15 or 16 or 17 or 18 or 19 or 20 or 21 or 22 or 23 or 24 or 25 or 26 or 27 or 28 or 29 or 30 or 31 or 32 or 33 or 34 or 35 or 36 or 37 or 38 or 39 353126

69 40 or 41 or 42 or 43 or 44 475646

70 45 or 46 or 47 or 48 or 49 or 50 or 51 or 52 or 53 or 54 or 55 or 56 or 57 or 58 or 59 or 60 or 61 or 62 or 63 or 64 or 65 or 66 or 67 1110035

71 68 and 69 and 70 530

72 limit 71 to english language 473

For peer review only

For peer review only

Record linkage studies of drug-related deaths among former adult prisoners who have been released to the community: a scoping review protocol

Cooper JA [1,2]*, Onyeka IN [1,2], O'Reilly D [1,2], Kirk R [3], Donnelly M [1,2]

[1] Centre for Public Health, Queen's University Belfast, Royal Hospitals Site, Grosvenor Road, Belfast, UK

[2] Administrative Data Research Centre Northern Ireland (ADRC NI), Centre for Public Health, Queen's University Belfast, Royal Hospitals Site, Grosvenor Road, Belfast, UK

[3] South Eastern Health and Social Care Trust, Ulster Hospital, Dundonald, UK

*corresponding author

For peer review only

DATA CHARTING FORM

**Study assessor initials**		
**Date of completion of data charting form**		
**Item**	***Notes on item***	**Data extracted**
**Study reference number**		
**Study assessment number**		
**Lead author**		

**Title**		
**Year of publication**		
**Origin, country of origin**		
**Journal name**		
**Study design**		
**Citation**		
**Setting**	*Stated setting*	
	Stated locations	
	Stated relevant dates	
**Participants**	*Stated study population*	
	Stated type of prison	
	Stated age inclusions (or any stated age exclusions)	
	Stated gender inclusions	
	Stated race/ethnicity inclusions	
	Stated methods of study population selection (type of prisoner data records)	
	Stated methods of follow-up (for example, record linkage or interview)	

For peer review only

	Stated rationale for the choice of cases and controls (case-control study only)	
	Stated matching criteria (case-control study only)	
	Stated number of controls per case (case-control study only)	
**Data sources**	*Stated sources of data*	
	Stated sources of comparator data	
**Bias**	*Stated bias*	
	Stated efforts to address potential sources of bias	
	Stated methods for dealing with repeated incarcerations?	
**Study size**	*Stated study size*	
**Statistical methods**	*Stated statistical methods*	
	Stated methods controlling for confounders and methods for examining subgroups and interactions	
	Stated sensitivity analyses	
	Stated time period examined after prison release	
	Stated how missing data was addressed	

For peer review only

	Stated how loss to follow-up was addressed	
	Stated how matching of case and controls was addressed (case-control study only)	
**Linkage**	*Stated linkage i.e. if person-level, institution-level or other data linkage across two or more databases*	
	Stated types of linked databases	
	Stated methods of linkage	
**Main outcomes**	*Stated outcome events or summary measures*<br>*(include information on specific drugs)*	
**Main results**	*Stated unadjusted estimate and, if applicable, confounder adjusted estimates, their precision, which confounders were adjusted for.*<br><br>*Reported for all cause and drug-related deaths*<br><br>*For example, include crude mortality rates, standardised mortality ratios etc.*<br><br>*Include reported for age, gender, race/ethnicity etc*	

For peer review only

	Stated age at release (mean/median/SD/range)	
	Stated length of incarceration (mean/median/SD/range)	
**Limitations**	*Stated limitations of study including sources of potential bias or imprecision (are the results generalisable)*	
**Quality assessment checklist or technique**	*Stated if quality assessment checklist or technique was used*	

For peer review only
